# Neuroprotective Fragment C of Tetanus Toxin Modulates IL-6 in an ALS Mouse Model

**DOI:** 10.3390/toxins12050330

**Published:** 2020-05-17

**Authors:** Laura Moreno-Martinez, Miriam de la Torre, María J. Muñoz, Pilar Zaragoza, José Aguilera, Ana C. Calvo, Rosario Osta

**Affiliations:** 1 Laboratory of Genetics and Biochemistry (LAGENBIO), Faculty of Veterinary, Institute for Health Research Aragon (IIS Aragón), AgriFood Institute of Aragon (IA2), Centro de Investigación Biomédica en Red sobre Enfermedades Neurodegenerativas (CIBERNED), University of Zaragoza, Miguel Servet 177, 50013 Zaragoza, Spain; lauramm@unizar.es (L.M.-M.); mtorre@unizar.es (M.d.l.T.); mjmunoz@unizar.es (M.J.M.); pilarzar@unizar.es (P.Z.); 2 Institut de Neurociències and Departament de Bioquímica i de Biologia Molecular, Facultat de Medicina, Universitat Autònoma de Barcelona (UAB) and Centro de Investigación Biomédica en Red sobre Enfermedades Neurodegenerativas (CIBERNED), 08193 Barcelona, Spain; Jose.Aguilera@uab.cat

**Keywords:** TTC, amyotrophic lateral sclerosis, inflammation, SOD1G93A mouse model

## Abstract

Neuroinflammation plays a significant role in amyotrophic lateral sclerosis (ALS) pathology, leading to the development of therapies targeting inflammation in recent years. Our group has studied the tetanus toxin C-terminal fragment (TTC) as a therapeutic molecule, showing neuroprotective properties in the SOD1G93A mouse model. However, it is unknown whether TTC could have some effect on inflammation. The objective of this study was to assess the effect of TTC on the regulation of inflammatory mediators to elucidate its potential role in modulating inflammation occurring in ALS. After TTC treatment in SOD1G93A mice, levels of eotaxin-1, interleukin (IL)-2, IL-6 and macrophage inflammatory protein (MIP)-1 alpha (α) and galectin-1 were analyzed by immunoassays in plasma samples, whilst protein expression of caspase-1, IL-1β, IL-6 and NOD-, LRR- and pyrin domain-containing protein 3 (NLRP3) was measured in the spinal cord, extensor digitorum longus (EDL) muscle and soleus (SOL) muscle. The results showed reduced levels of IL-6 in spinal cord, EDL and SOL in treated SOD1G93A mice. In addition, TTC showed a different role in the modulation of NLRP3 and caspase-1 depending on the tissue analyzed. In conclusion, our results suggest that TTC could have a potential anti-inflammatory effect by reducing IL-6 levels in tissues drastically affected by the disease. However, further research is needed to study more in depth the anti-inflammatory effect of TTC in ALS.

## 1. Introduction

Amyotrophic lateral sclerosis (ALS) is a devastating neurodegenerative disease characterized by the loss of the cortex, brainstem, and spinal cord motor neurons (MNs). This motoneuron degeneration leads to muscle paralysis and, finally, premature death due to respiratory failure within 2–5 years after diagnosis. There is currently no cure for the disease, although some drugs are used to slow disease progression, such as riluzole, and more recently, edaravone and masitinib [1]. Despite the numerous pathologic mechanisms proposed, the etiology of the majority of ALS cases—sporadic ALS (sALS)—is unknown.

Neuroinflammation is a relevant hallmark in ALS pathology and is characterized by microglial activation, astrogliosis, infiltration of T lymphocytes and monocytes, and overproduction of inflammatory cytokines [2,3,4]. It has been suggested that inflammatory factors may modulate the disease progression and survival rate in ALS. Accordingly, a wide range of pro-inflammatory mediators, such as interleukins, have been deeply studied in recent years with the purpose of identifying a panel, useful to predict the disease course. The interleukins most frequently found dysregulated in ALS were interleukin (IL)-2, IL-6 and IL-17 [5], although there are some controversial results that question the role of these cytokines as reliable biomarkers. More recently, another mechanism associated with the immune system, the NLRP3 inflammasome, has been studied in ALS, showing its involvement in the disease [6,7,8]. NLRP3 inflammasome is an essential mechanism involved in immune responses through the activation of caspase-1 leading to the maturation of IL-1β and IL-18, and the induction of pyroptosis [9]. The cause of the participation of NLRP3 inflammasome in ALS could underlie in the misfolded protein aggregates found in ALS, as they could become damage-associated molecular patterns (DAMPs) and cause direct activation of NLRP3, following the activation of cytokines [10]. In an attempt to modulate the inflammatory mechanisms in ALS, therapies targeting inflammation have emerged in recent years showing promising results, such as the infusion of regulatory T cells (Tregs) [11,12,13].

In view of this, it becomes vital to find therapies aimed to control the dysregulation of the immune system in ALS to mitigate the inflammatory responses that result harmful in the disease. Our group has been studying the potential role of the tetanus toxin C-terminal fragment (TTC) as a therapeutic molecule in ALS during the last decade. Its non-toxic nature and neuroprotective properties have made TTC a potential carrier molecule through the nervous system as well as a therapeutic molecule for neurological disorders [14,15]. Especially in the SOD1G93A mouse model, we demonstrated that naked DNA encoding for TTC treatment ameliorated the decline of hindlimb muscle innervation, significantly delayed the onset of symptoms and functional deficits, improved spinal motor neuron survival, and prolonged lifespan [16]. In addition, our previous in vitro study with the TTC protein have shown similar results [17].

Despite the participation of TTC in several mechanisms dysregulated in ALS, such as apoptosis, it is unknown whether TTC could have some effect at the immunological or inflammatory level. Thus, the aim of this work was to study the possible anti-inflammatory effect of TTC in the SOD1G93A mouse model by studying its effect on the regulation of some pro-inflammatory cytokines previously described altered in ALS, and on NLRP3 inflammasome proteins.

## 2. Results

### 2.1. TTC Did Not Reduce Any Cytokine in Plasma from SOD1G93A Mice 

**Figure 1:**
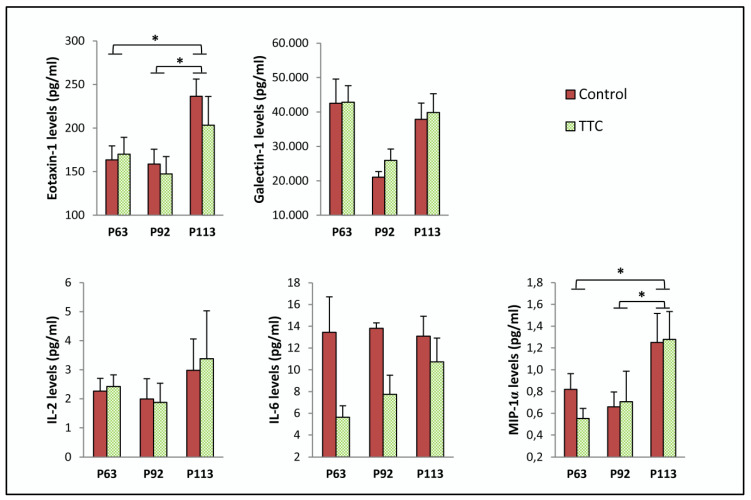
Expression of pro-inflammatory mediators in serial plasma samples from control and TTC-treated SOD1G93A transgenic mice at P63, P92 and P113. The sample size was 11 mice (5 control and 6 treated with tetanus toxin C fragment protein (TTC)) for eotaxin-1, interleukin (IL)-2, IL-6 and macrophage inflammatory protein (MIP)-1 alpha (α), and 22 mice (11 control and 11 TTC) for galectin-1. Two-way ANOVA with Bonferroni post hoc test was used. Graphics shows mean ± standard error of the mean. * *p* < 0.05.

Protein levels of eotaxin-1, galectin-1, IL-2, IL-6 and macrophage inflammatory protein (MIP)-1 alpha (α) were analyzed in serial plasma samples from SOD1G93A mice under TTC treatment and their controls (Table 1). We did not observe statistically significant differences in the levels of any cytokine measured when comparing the two groups of mice. However, levels of eotaxin-1 and MIP-1α were significantly higher at P113 comparing to the levels found at P63 and P92, regardless of treatment. (Figure 1).

### 2.2. TTC Differently Modulates the Expression of NLRP3, Caspase-1 and IL-6 in Spinal Cord and Two Types of Skeletal Muscle Fibers from SOD1G93A Mice 

**Figure 2:**
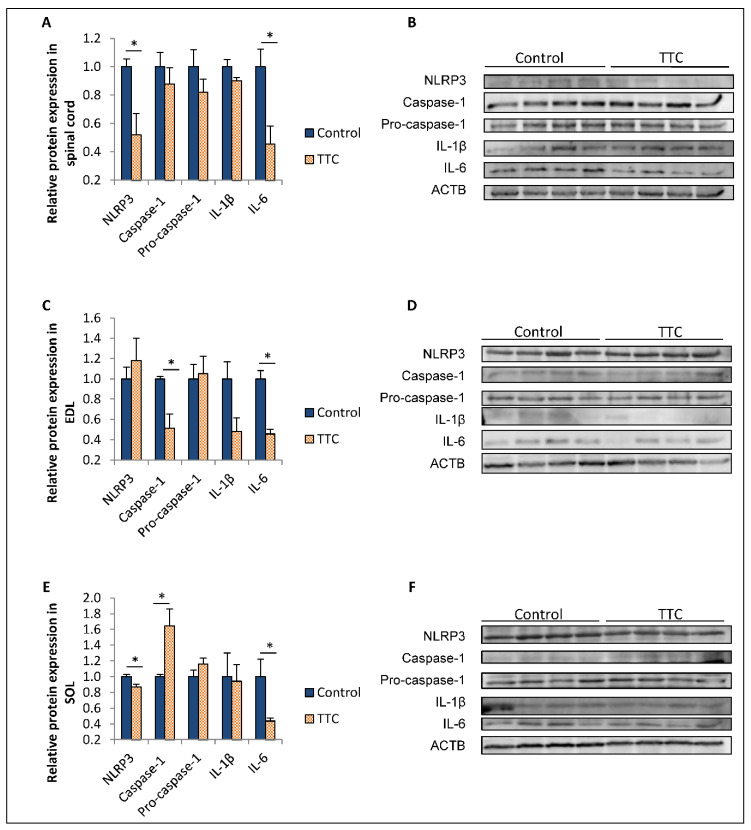
Relative protein expression of NLRP3, caspase-1, pro-caspase-1, IL-1β and IL-6 in spinal cord, EDL and SOL from control and TTC-treated SOD1G93A transgenic mice at P113. Protein expression of NOD-, LRR- and pyrin domain-containing protein 3 (NLRP3) (106 kDa), caspase-1 (20 kDa), pro-caspase-1 (45 kDa), interleukin (IL)-1β (31 kDa) and IL-6 (26 kDa) was analyzed in spinal cord (**A**,**B**), extensor digital longus (EDL) muscle (**C**,**D**) and soleus (SOL) muscle (**E**,**F**). The housekeeping protein selected to normalize the results was actin beta actin (ACTB) (42 kDa). The sample size was 4 control and 4 mice treated with tetanus toxin C fragment protein (TTC). Unrelated *t*-test or Mann–Whitney *U*-tests were used. Graphics shows relative mean ± standard error of the mean. * *p* < 0.05.

Caspase-1, IL-1β, IL-6 and NOD-, LRR- and pyrin domain-containing protein 3 (NLRP3) protein expression was quantified in the spinal cord, extensor digitorum longus (EDL) muscle and soleus (SOL) muscle from SOD1G93A mice treated with TTC and their controls at a late stage of the disease (Figure 2). The western blot assay showed that the levels of IL-6 and NLRP3 in the spinal cord and SOL were decreased in the group of mice under TTC treatment compared to their controls. Similarly, we found a downregulation of IL-6 in EDL in the TTC-treated group of mice. In contrast, we could observe different effect of TTC depending on the muscle. NLRP3 protein levels were not found decreased in EDL from the TTC-treated group. On the other hand, caspase-1 was found decreased in EDL and increased in SOL from mice treated with TTC.

## 3. Discussion

Neuroinflammation is a significant feature of ALS pathogeny. Therefore, the development of therapies that can modulate the inflammatory mechanisms arisen in ALS could be helpful in the fight with this disease. Previous studies have demonstrated the positive effect of TTC in the SOD1G93A mouse model by protecting against spinal motor neuron loss, reducing microgliosis, delaying the onset of symptoms and prolonging lifespan [15,16]. However, whether TTC could modulate the inflammation occurring in ALS is unknown. In this work, we studied the effect of a TTC-based therapy in the SOD1G93A transgenic mice on the regulation of several pro-inflammatory mediators in plasma, and also on NLRP3 inflammasome proteins in blood and two of the most affected tissues in the disease: spinal cord and skeletal muscle.

IL-6 levels were measured in serial plasma samples from control and TTC-treated transgenic mice at three different stages of the disease. Concurrently, IL-6 protein expression was analyzed in spinal cord, EDL and SOL from these mice at a late stage of the disease. Our results showed a consistent downregulation of IL-6 in the spinal cord, EDL and SOL muscles from mice which received TTC injections. However, we did not observe this effect in blood. IL-6 is a cytokine involved in inflammation and the maturation of B cells, and it is principally produced at sites of acute and chronic inflammation. In relation to ALS, many studies have reported higher levels of IL-6 in blood from ALS patients compared to healthy patients or in ALS mouse models [18,19,20,21,22,23,24,25]. However, there are works pointing out the large variability of this cytokine in blood [26,27], highlighting that its dysregulation in this tissue does not seem to be specific to ALS [28,29]. Regarding the skeletal muscle, a previous study found higher levels of IL-6 in the skeletal muscle of SOD1G93A mice compared to WT mice [30]. Therefore, our findings could imply that TTC may be helpful in counteracting the inflammatory mechanisms promoted by the activation of IL-6 in the most affected tissues in ALS, such as the skeletal muscle and the spinal cord. Despite the large variability observed in the literature, the alteration of IL-6 in ALS patients and ALS animal models along disease course is frequent [5,22]. Given that elevated levels of IL-6 can lead to a higher status of inflammation, strategies aimed at reducing this cytokine could be useful to modulate inflammation occurring in ALS. In this sense, several therapies showing some positive effects in the disease were also able to modulate IL-6 expression, suggesting that this modulation could help in the improvement of ALS pathology [31,32,33]. According to this, our finding showing the effect of TTC downregulating the expression of IL-6 in spinal cord and skeletal muscle may suggest that TTC could have a role in palliating the disease course through reducing inflammation mediated by IL-6.

Apart from IL-6, other pro-inflammatory mediators were measured in serial plasma samples from the transgenic mice: eotaxin-1, galectin-1, IL-2 and MIP-1α. The selection of these cytokines was based on a previous study where we found negative correlations between the levels of these proteins in plasma and the survival rate of the SOD1G93A transgenic mice [25]. Accordingly, other studies reported increased levels of eotaxin-1, IL-2 and MIP-1α in plasma from ALS patients compared to healthy patients [20,34,35,36]. However, we could not observe any difference in the levels of any of these proteins between the control group and the TTC-treated group. Although reducing these mediators could result in interest to elucidate their role in ameliorating the disease course, TTC did not seem to exert an effect on the modulation of the levels of these cytokines in plasma. Nevertheless, we found that the age of the mice had an effect in the expression of eotaxin-1 and MIP-1α, regardless the treatment received, revealing higher levels at the terminal stage. These results are in line with our previous work regarding eotaxin-1 [25].

Expression of proteins belonging to NLRP3 inflammasome, particularly caspase-1, IL-1β and NLRP3, was analyzed by western blot in spinal cord, EDL and SOL. We could observe different effects exerted by TTC depending on the tissue. NLRP3 was downregulated in both spinal cord and SOL from TTC-treated mice; in contrast, this significant change was not found in EDL. Regarding caspase-1, we found a different regulation of TTC depending on the muscle: it was decreased in EDL, whereas it was upregulated in SOL. IL-1β was also studied, but we could not find any change dependent on the TTC treatment. The contribution of NLRP3 inflammasome and its components in ALS pathogeny has been studied in recent years [7,10,37]. For instance, it has been demonstrated to be activated in brain of SOD1G93A rats through an upregulation of the protein levels of NLRP3, caspase-1, IL-1β and IL-18 compared to wildtype (WT) rats [8]. Similarly, it was found activated also in spinal cords from SOD1G93A mice and ALS patients [38]; specifically, NLRP3 was found increased only at a late stage of the disease in transgenic mice. According to our results, TTC reduced NLRP3 levels in spinal cord at P113, suggesting that TTC may modulate the late activation of NLRP3 in this tissue. On the other hand, lower caspase-1 gene expression in spinal cord in non-treated SOD1G93A mice was already described by our group, which is in accordance with our results [16]. NLRP3 inflammasome is also active in the skeletal muscle, showing increased levels of caspase-1 and IL-1β in pre-symptomatic SOD1G93A mice, and in denervated muscles from these mice and ALS patients [39]. According to our results, TTC could have a role in palliating the effects of active caspase-1 in EDL, but not in SOL. The differences observed between both skeletal muscles could be explained by their opposed phenotypes: slow-twitch SOL and fast-twitch EDL, as depending on their fiber-composition, they can show different expression patterns of myokines or other proteins [40,41]. In this sense, the protective effect of TTC could modulate caspase-1 levels only in EDL, which is mostly affected by the disease progression with respect to SOL.

Given the evident activation of the NLRP3 inflammasome in ALS, therapies targeting that activation have been developed in an attempt to palliate the inflammatory processes. In ALS microglial cells, NLRP3 inflammasome activation was inhibited through the anti-inflammatory cyclic dipeptide (His-Pro) [42]. Similarly, the anti-inflammatory molecule 17β-estradiol improved the motoneurons survival in SOD1G93A and reduced the NLRP3 inflammasome in the spinal cord of these mice, especially caspase-1 and IL-1β, suggesting the potential beneficial effect of modulating NLRP3 inflammasome in ALS [43]. In line with these findings, we observed that TTC could modulate the levels of NLRP3 and caspase-1 in spinal cord, EDL and SOL. However, these changes did not imply the following decrease in the other components of the inflammasome, especially IL-1β, which is the final effector of this complex and finally leads to neuroinflammation. Further research is needed to elucidate to what extent these changes could lead to an alleviation of the mechanisms involving NLRP3 inflammasome in the disease.

## 4. Conclusions

Our results suggest that TTC could have a potential anti-inflammatory effect by reducing IL-6 levels in tissues drastically affected by the disease, and also, modulating the expression of NLRP3 inflammasome proteins. Although further research is needed to study more in depth the anti-inflammatory effect of TTC in ALS, this characteristic added to the other properties of TTC previously observed in the SOD1G93A mice may point out TTC as a potential therapeutic molecule for ALS.

## 5. Materials and Methods 

### 5.1. Animals

Transgenic mice were obtained by mating hemizygous B6SJL-Tg SOD1G93A males (stock number 002726) purchased from The Jackson Laboratory (Bar Harbor, ME, USA) with C57BL/6J × SJL/J F1 hybrid females (B6SJLF1) purchased from Janvier Labs (Saint-Berthevin Cedex, France). The offspring were identified by PCR assay as described in The Jackson Laboratory protocol. The mice were housed at the animal facilities in Centro de Investigación Biomédica de Aragón (CIBA) under a standard light:dark (12:12) cycle. Food and water were provided ad libitum. 

All procedures were approved by the Ethic Committee for Animal Experiments from the University of Zaragoza. The care and use of animals were performed accordingly with the Spanish Policy for Animal Protection RD53/2013, which meets the European Union Directive 2010/63 on the protection of animals used for experimental and other scientific purposes. The approval code is the PI14/17 and the approval date: 21 April 2017.

### 5.2. Treatment with Recombinant TTC Protein

Recombinant TTC protein was kindly provided by José Aguilera from UAB, CIBERNED, Barcelona, Spain. Twelve SOD1G93A mice were injected via intramuscular with 1 μg of TTC recombinant protein at P59, corresponding to the early symptomatic stage. Simultaneously, twelve SOD1G93A mice were injected with phosphate-buffered saline (PBS) as the control group. Groups were sex-balanced (6 males and 6 females in each group) and littermates were equally distributed in both groups. Injections were made weekly until P113, when they were euthanized to proceed to collect the samples. A treatment scheme can be found in the supplementary data, Table S1.

### 5.3. Sample Collection

Serial plasma samples were taken from the twenty-four mice at three stages: 63, 92 and 113 days of postnatal life (P63, P922 and P1133), as they correspond to the early symptomatic, late symptomatic and terminal stages, respectively [44,45,46]. Plasma samples at P63 and P92 were obtained from submandibular bleeding, whilst those at terminal stage were collected from blood extracted by cardiac puncture immediately after euthanasia. Blood was extracted and transferred to EDTA coated mini vacutainer tubes (BD Biosciences, San Jose, CA, USA). Then, blood was centrifuged at 3000 rpm for 10 min at 4 °C within 30 min from extraction, plasma was collected and immediately frozen in dry ice and stored at −80 °C.

Spinal cord and two different skeletal muscles, EDL and SOL, were isolated from SOD1G93A mice after euthanized with CO_2_ at P113. These tissues were immediately frozen in dry ice and stored at −80 °C until processed. 

### 5.4. ELISA and Multiplex Immunoassays

Levels of eotaxin-1, IL-2, IL-6 and macrophage inflammatory protein (MIP)-1 alpha (α) were analyzed by multiplex immunoassay (ProcartaPlex Multiplex Immunoassay, Affymetrix eBioscience, Thermo Fisher Scientific Inc., Waltham, MA, USA ) and galectin-1 levels by enzyme-linked immunosorbent assay (ELISA) (Boster Biological Technology, Pleasanton, CA, USA) in serial plasma samples from eleven (in multiplex) and twenty two (in ELISA) control and TTC-treated SOD1G93A mice at the three different stages of the disease. 

### 5.5. Western Blotting

Spinal cord, EDL and SOL from eight control and TTC-treated SOD1G93A mice were ground into a powder using the Tissue Lyser LT (Qiagen, Germantown, MD, USA). Then, powdered tissues were resuspended in RIPA lysis buffer together with protease inhibitors (SC-24948, Santa Cruz Biotechnology, Inc., Santa Cruz, CA, USA) according to manufacturer’s protocol. Total protein was quantified using the BCA method (Sigma-Aldrich, St. Louis, MO, USA). A total of 30 μg of protein was loaded into a 10% SDS-PAGE gel to analyze the protein expression of caspase-1, IL-1β, IL-6 and NLRP3. After electrophoresis, proteins were transferred to a PVDF membrane (Amersham™, GE Healthcare Life Sciences, Marlborough, MA, USA) and subsequently blocked with a Tris-buffered saline solution containing 5% bovine serum albumin (BSA) and 0.1% Tween as supplement for 1 h at room temperature. Then, membranes were incubated overnight at 4 °C with the following primary antibodies: caspase-1 (sc-1218-R, Santa Cruz Biotechnology, Inc., Santa Cruz, CA, USA), IL-1β (sc-7884, Santa Cruz Biotechnology, Inc., Santa Cruz, CA, USA), IL-6 (PK-AB815-61632M, Promocell, Heidelberg, Germany) and NLRP3 (sc-66846, Santa Cruz Biotechnology, Inc., Santa Cruz, CA, USA). The housekeeping protein selected to normalize the results was actin beta actin (ACTB) (PA1-183, Thermo Fisher Scientific, Waltham, MA, USA). Secondary antibody used was goat anti-Rabbit IgG (31466, Thermo Fisher Scientific, Waltham, MA, USA). Finally, chemiluminescence detection was performed using Immobilon Crescendo Western HRP Substrate (Millipore, Billerica, MA, USA) in a Molecular Imager^®^ VersaDoc™ MP 4000 system. The computer-assisted analysis of the bands was performed with AlphaEase FC software (Bonsai Technologies Group, S.A., Madrid, Spain). 

### 5.6. Statistical Analysis

Results obtained in immunoassays were compared between the treated and control SOD1G93A groups using two-way analysis of variance ANOVA with Bonferroni post hoc test, as the distribution of the data was normal according Shapiro-Wilk test. Results obtained in western blot were compared between the treated and control SOD1G93A groups using *t*-tests or Mann–Whitney *U*-tests, according to data distribution analyzed by Shapiro-Wilk test. Statistical analysis was performed using SPSS (version 20, IBM, Armonk, NY, USA) and GraphPad Prism Software (version 5, La Jolla, CA, USA). All of the values were expressed as the mean ± standard error of the mean (SEM). Significance levels were set at *p* < 0.05.

## Figures and Tables

**Table 1:** Levels of pro-inflammatory mediators in serial plasma samples from control and TTC-treated SOD1G93A transgenic mice at P63, P92 and P113.

Protein	Age	Control	TTC	*p*-Value
Eotaxin-1 (pg/mL)	P63	163.407 ± 15.992	170.119 ± 19.104	0.799
	P92	158.566 ± 17.151	147.400 ± 19.803	0.687
	P113	236.529 ± 19.625	203.274 ± 33.074	0.412
Galectin-1 (pg/mL)	P63	42,562.638 ± 6977.809	42,872.392 ± 4757.342	0.971
	P92	21,041.705 ± 1657.878	25,930.396 ± 3328.63	0.192
	P113	37,897.955 ± 4724.214	39,839.61 ± 5479.457	0.798
IL-2 (pg/mL)	P63	2.262 ± 0.442	2.420 ± 0.403	0.816
	P92	1.994 ± 0.697	1.870 ± 0.667	0.902
	P113	2.980 ± 1.077	3.376 ± 1.654	0.853
IL-6 (pg/mL)	P63	13.451 ± 3.255	5.626 ± 1.064	0.062
	P92	13.810 ± 0.502	7.735 ± 1.764	* 0.021
	P113	13.097 ± 1.828	10.727 ± 2.188	0.429
MIP-1α (pg/mL)	P63	0.820 ± 0.144	0.552 ± 0.093	0.157
	P92	0.659 ± 0.137	0.706 ± 0.281	0.877
	P113	1.250 ± 0.267	1.278 ± 0.256	0.941

Note: TTC: tetanus toxin C-terminal fragment; * *p* < 0.05

## Supplementary Materials

The following are available online at https://www.mdpi.com/2072-6651/12/5/330/s1, Table S1: Scheme of the timeline followed for the treatment of TTC and the sample collection.
