# Supplementary material for: Neuroprotective Fragment C of Tetanus Toxin Modulates IL-6 in an ALS Mouse Model
Source: Toxins (Basel). 2020 May 17;12(5):330. doi: 10.3390/toxins12050330 (PMC7290364; doi:10.3390/toxins12050330)
Supplement: Supplementary file 1 [file toxins-12-00330-s001.pdf]

Supplementary Materials: Neuroprotective Fragment C of Tetanus Toxin Modulates IL-6 in an ALS Mouse Model

Laura Moreno-Martinez, Miriam de la Torre, María J. Muñoz, Pilar Zaragoza, José Aguilera, Ana C. Calvo and Rosario Osta

Table S1. Scheme of the timeline followed for the treatment of TTC and the sample collection.

**Age of Mice (days)**	**59**	**63**	**66**	**73**	**80**	**87**	**92**	**94**	**101**	**108**	**113**
**Event**	Start of TTC treatment	Blood collection	TTC treatment	TTC treatment	TTC treatment	TTC treatment	Blood collection	TTC treatment	TTC treatment	End of TTC treatment	Sacrificed and sampling

Note: TTC: tetanus toxin C-terminal fragment.
